# Impacts of Cold Plasma Technology on Sensory, Nutritional and Safety Quality of Food: A Review

**DOI:** 10.3390/foods11182818

**Published:** 2022-09-13

**Authors:** Bo Zhang, Chunming Tan, Fanglei Zou, Yu Sun, Nan Shang, Wei Wu

**Affiliations:** 1 College of Engineering, China Agricultural University, Beijing 100083, China; 2 Beijing Laboratory for Food Quality and Safety, College of Food Science and Nutritional Engineering, China Agricultural University, Beijing 100083, China; 3 Key Laboratory of Precision Nutrition and Food Quality, Department of Nutrition and Health, China Agricultural University, Beijing 100083, China

**Keywords:** cold plasma, plasma species, organoleptic properties, nutrient content, safety performance

## Abstract

As an emerging non-thermal food processing technology, cold plasma (CP) technology has been widely applied in food preservation due to its high efficiency, greenness and lack of chemical residues. Recent studies have indicated that CP technology also has an impressing effect on improving food quality. This review summarized the impact of CP on the functional composition and quality characteristics of various food products. CP technology can prevent the growth of spoilage microorganisms while maintaining the physical and chemical properties of the food. It can maintain the color, flavor and texture of food. CP can cause changes in protein structure and function, lipid oxidation, vitamin and monosaccharide degradation, starch modification and the retention of phenolic substances. Additionally, it also degrades allergens and toxins in food. In this review, the effects of CP on organoleptic properties, nutrient content, safety performance for food and the factors that cause these changes were concluded. This review also highlights the current application limitations and future development directions of CP technology in the food industry. This review enables us to more comprehensively understand the impacts of CP technology on food quality and promotes the healthy application of CP technology in the food industry.

## 1. Introduction

With the increasing demand of consumers for high-quality products, the food industry is looking for new technologies that can improve product safety, extend shelf life and maximize the quality of food. Heat treatment is a commonly used and relatively economical technology in the food industry, which may destroy the flavor, color, texture and nutrients of heat-sensitive foods [1]. In recent years, non-thermal technology as a new technology has been widely applied in the food industry because of its low processing temperature and because it can avoid the harm caused by thermal processing technology [2]. Non-thermal processing technologies in food processing mainly include ozone treatment, pulsed electric field, ultrasonic treatment, ionizing radiation, cold plasma (CP) treatment and so on.

CP, as an emerging non-thermal processing technology, can efficiently improve food safety and quality. It is becoming more and more popular due to its high efficiency, environmental protection and lack of chemical residues [3]. CP is usually generated by the discharge of air or inert gases such as nitrogen, helium and argon under vacuum or atmospheric pressure [4]. The active plasma species (such as active nitrides and active oxides) produced during its processing can effectively kill microorganisms on the food surface and cause changes in the functional characteristics of the food. CP has been used in many areas of the food industry, such as food microbiological purification [5,6], the degradation of pesticides and toxins in food [7,8,9] and the surface treatment of food packaging materials [10,11]. However, research on the effects of CP treatment on food functional components and quality characteristics is still in the preliminary stage. Therefore, this review aims to briefly describe the impact of CP treatment on the quality of food in recent years.

## 2. Fundamentals of Cold Plasma Technology

**Figure 1:**
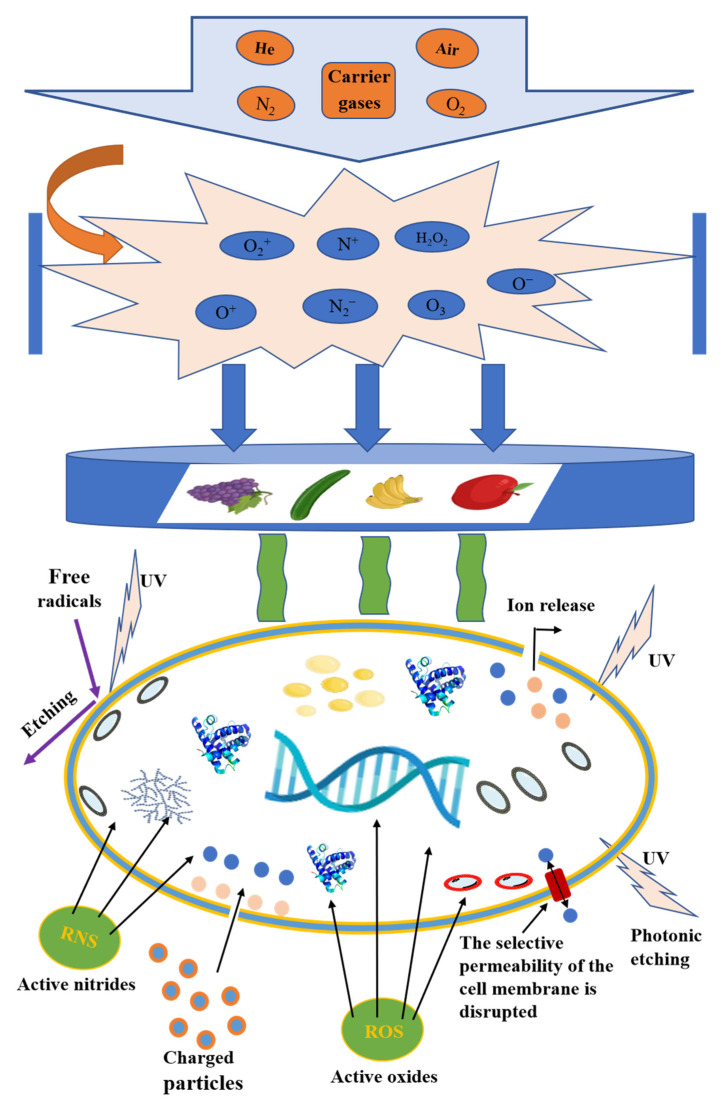
The generation process and action mechanism of plasma.

Plasma, called the fourth state of matter, is a completely or partially ionized gas mixture consisting of photons, electrons, positive and negative ions, neutral particles and free radicals [12]. It is generated by applying exciting energy (e.g., thermal energy, an energetic beam or an electric field) through neutral gas (e.g., air, oxygen or nitrogen), which leads to the dissociation of the gaseous molecules and the ionization of atoms. A typical plasma generation process is shown in Figure 1. When the gas is energized, it produces a sea of reactive oxygen (such as O_2_^+^, O^−^, O^+^, O_3_ and H_2_O_2_) and reactive nitrogen (such as N_2_^*^, N•, N^+^, N_2_^+^, N^*^ and NO), which reacts with the compounds in the food and changes the quality of the food [13]. The number of positive and negative charges in the gas is always equal numerically, so it behaves electrically neutral [14]. Based on its thermodynamic equilibrium, it is divided into thermal or non-thermal plasma. Atmospheric pressure non-thermal plasma, also known as “cold plasma”, has electrons with high energy, while other particles, such as ions and neutrons, can keep a relatively low temperature. CP can be generated at room temperature and atmospheric pressure. On the one hand, its electrons have high enough energy to excite and ionize the reactants, and on the other hand, the reaction system remains at a low temperature, which can reduce energy consumption, so it is widely applied to the food industry.

### 2.1. Generation of Cold Plasma

In general, CP can be generated by various exciting energies such as thermal energy, an energetic beam and an electric field [15]. The carrier gases that can be used in the CP technology are usually oxygen, nitrogen, atmospheric air, noble gases or a combination of these gases. Air as the carrier gas has been proved to be a cost-effective working gas and has been widely used in the food industry.

**Figure 2:**
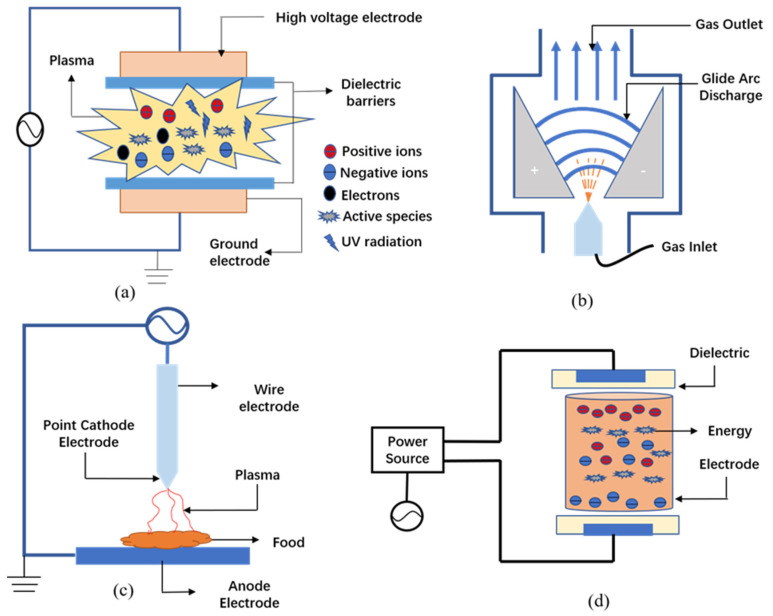
Schematic diagram of various equipment for generating plasma [16]. (**a**) dielectric barrier discharge, (**b**) corona discharge, (**c**) glow discharge, (**d**) glide arc discharge.

In recent years, a variety of plasma generation equipment has been produced for targeting the production needs of different fields. The generating systems often used in CP include dielectric barrier discharges, corona discharge, glow discharge and glide arc discharge. These papers comprehensively record various plasma generating equipment. Figure 2 depicts a schematic diagram of various equipment for generating plasma [16].

Dielectric barrier discharge (DBD) reactors are mostly connected to a high-voltage AC power supply, and the reactor includes three parts: a high-voltage electrode, ground electrode and dielectric. In DBD devices, two electrodes covered by dielectric (such as glass, plastic, silicon or ceramic) are used for plasma generation. Preventing the generation of electric sparks and thermal plasma is the role of the insulators [17]. The operating frequency of DBD is generally between 0.05 and 500 kHz, and gas pressure is generally between 104–106 Pa [18]. DBD usually has three forms: single-sided dielectric barrier discharge, double-sided dielectric barrier discharge and intermediate dielectric barrier discharge. DBD technology is one of the most convenient forms of plasma generation. Due to its low industrial cost and relatively flexible electrode shape, DBD is increasingly widely used in the field of food processing.

Glide arc discharge (GAD) is generally generated at a short distance between two electrodes. Propelled by the gas, the arc moves along the electrode. When the length of the sliding arc reaches the critical length, the power supply cannot provide enough energy to balance the heat loss. The sliding arc is rapidly extinguished at this stage, and the arc is reformed in the narrowest interelectrode area [19]. GAD has positive effects on both liquid treatment and surface treatment and has been used in different fields such as material surface modification, wastewater treatment and decomposition of inorganic pollutants.

The conditions for corona discharge (CD) are: high gas pressure, uneven electric field distribution and a voltage of several thousand volts or more applied to the electrode. Therefore, a non-uniform electric field will form between point and point, tip and plane, two parallel electrical wires and a corona may form between these electrodes. CD occurs in a highly uneven electric field and appears in the form of luminescence in the space around the point tip [20]. Ion flow, light radiation and neutral molecular flow are the main ways for the energy of CD to act on the surface of the material. The system can work in pulse voltage or DC current mode and does not require complicated equipment and high operating costs. The main disadvantage of this system is the non-uniformity of the treatment [21]. CD has been used in many industries such as surface treatment, microbial purification, etc. 

After crossing the CD area, if the resistance of the external circuit is reduced, or the voltage of the whole circuit is increased, and the discharge power continues to increase, the discharge current will continue to rise. The glow gradually expands into the discharge space between the two electrodes, and the light becomes brighter and brighter, which is called glow discharge (GD). GD is generated by applying high voltage electricity to a low-pressure gas, which can generate plasma at large volumes and low temperatures [17]. At least one electrode in this system needs to be covered by an insulating medium. The charged particles will gather on the surface of the insulator after the power is turned on, forming a potential difference between the insulating media.

### 2.2. Factors Influencing the Efficiency of Cold Plasma

#### 2.2.1. Internal Factors

The internal factors that affect the efficiency of CP processing include the type of processing gas, processing voltage and frequency, plasma flow rate and so on. The type of carrier gas determines the chemically reactive species generated in food processing. Air, nitrogen, oxygen and inert gases such as helium and argon, or a combination thereof, are often selected as working gases in CP technology. Air is currently the most widely used and lowest cost carrier gas. Air produces numerous reactive nitrides and reactive oxides during the reaction. These reactive species can react with organic compounds within cells. Due to the stable nature of inert gas, it changes little in the chemical composition of food [22]. The use of inert gases in combination with oxygen can reduce the oxidative degradation of food chemicals. Ozone, NxOy and peroxy-radicals are formed when a nitrogen and oxygen mixture is used for discharge. When water is present in the reaction gas, it leads to the formation of OH, H species and H_2_O_2_ [23]. Similarly, the quantity of the plasma species produced during the reaction is affected by the input voltage and frequency [24]. There are several reports on the effects of different processing voltages and frequencies on food composition [25,26,27]. Another process variable which can affect CP efficiency is the flow rate of the plasma. Some reactive species with a short half-life may not be able to react with food at low flow rates while a faster gas flow rate can enhance the reaction probability to a certain extent [18]. Butscher, et al. [28] reported that the increased transfer rate of plasma species to the sample surface improved the inactivation effect of plasma on microorganisms.

#### 2.2.2. External Factors

The factors such as pH, the matrix of the sample, relative humidity, time and exposure conditions greatly affect the efficiency of CP treatment. For example, solid and liquid food substrates interact differently with reactive substances during CP processing. In the plasma treatment process, the penetration depth of chemically reactive species is different for solid and liquid food. In liquid food, all the components can be fully in contact with the plasma species. However, for solid food matrixes, the composition, porosity and moisture content of the food can affect the penetration rate of the active particles [29]. pH is another pivotal external factor for CP treatment to achieve the desired effect. Muranyi, et al. [30] reported that the reduction in Bacillus cereus with different pH was discrepant after CP treatment, and the decrement of *Bacillus cereus* with a low pH was more distinct. In addition, the increase in humidity (the humidity of the gas phase or the water content of the food) in the plasma environment can produce more different active substances, such as hydrogen peroxide, perhydroxyl radicals, superoxide anions and other ROS, which improves the oxidation and antibacterial effects [29].

## 3. The Effect of CP on Organoleptic Properties of Foods

Organoleptic properties are the most direct attributes of food, which can directly affect the overall acceptance of the product. A large number of studies have shown that CP treatment will affect the color, structure and flavor of food. Table 1 summarized some research on the effect of CP treatment on the organoleptic properties of food in recent years.

### 3.1. Color

Color is one of the most important sensory characteristics of food, and it can directly affect the acceptance of the product. Pigments (natural or synthetic) and chemical reactions during CP processing may change the color of food.

Numerous studies have observed the color of CP-treated food. Ali et al. [31] found that with the increase in treatment time, the difference in the total color value of tomato juice increased significantly, which might be due to the decomposition of carotenoid pigments by plasma species. CP treatment significantly changed the color of apple juice, and the juice became brighter and yellower [32]. This change was related to oxidation reaction and pigment isomerization. After CP treatment, the color of coconut juice, ready-to-eat sliced chicken sausage and pumpkin puree also changed significantly [47,48,49]. However, some scholars have observed results that were inconsistent with the research mentioned above. For example, in some studies using CP to treat raised chicken, blueberry juice, strawberries and siriguela juice, no significant color changes were found in the samples [33,34,35,50]. In recent years, some scholars have discovered that CP treatment can replace traditional chemical agents (such as nitrite) to make meat products appear bright red [36,37]. In addition, Koddy, et al. [38] treated hairtail samples with ACP and found that the brightness value was higher than that of the control sample. The main reason for the improvement in the color of hairtail protein may be the increase in the water holding capacity.

In general, CP technology does not significantly change the color of food when treated for less than five minutes [51]. Plasma processing parameters (input power, voltage, carrier gas, exposure time) and food state (solid or liquid, whole or cut) are all key factors that affect the color of the product. As an effective green non-thermal treatment method, CP treatment can replace harmful chemical reagents to maintain and improve the color of meat products.

### 3.2. Texture

Texture is an essential indicator to evaluate the quality of food. It affects the taste of the product and it also determines whether the food can be stored and transported over long distances. The texture of food is closely related to its organizational structure.

Many studies have found that CP technology can improve the texture of fresh food. Gavahian, et al. [39] used DBD and plasma-activated water (PAW) to treat mushroom samples separately and found that they did not significantly change the hardness of the mushroom samples in a short period. After 1 week of storage, the plasma-treated samples had a higher hardness value than the control sample, and the PAW mushroom had the highest hardness value, indicating that plasma technology has potential application value in improving the shelf life and quality of food. In addition, Koddy, et al. [38] found that CP treatment can improve the texture performance of hairtails, which was attributed to CP treatment oxidizing hairtail muscle proteins and making the protein network more intensive. Similarly, Ma, et al. [44] reported a similar result. Another study found that CP technology can significantly change the surface morphology of jujube slices [52]. The change was correlated with the etching effect of plasma species, which makes the surface of jujube slices appear to have larger pores and accelerates the evaporation of water. Similar results have been found in some applications of CP treatment in the drying of peppers, okra and corn kernels [40,41,53]. However, other studies have found that the reduction in sample moisture by plasma may also be due to the reaction of plasma species with sample moisture. For example, Shirani et al. [42] observed that plasma treatment increases the hardness of almond slices. This is due to the reaction between the plasma species and the moisture in the almond flakes that causes the moisture to be converted into other compounds. Y. Chen et al. [43] also obtained similar results in the study of using plasma to dehydrate fresh and wet noodles. However, a decrease in firmness was reported after blueberry CP treatment [54,55]. This was attributed to the mechanical damage to the blueberry surface caused by the elevated temperature of the plasma jet and the high air velocity.

### 3.3. Flavor

Food flavor is also a necessary sensory characteristic for evaluating food quality. Chemical reaction products (such as aromatic compounds) during food processing can affect the flavor of food [54]. Studies have found that CP treatment produced peculiar odors in Asian sea bass fillets and reduced the overall acceptance, which may be attributed to the increased lipid oxidation rate of plasma species [46]. Similarly, Ke et al. [56] found that the main products of CP, RNS and ROS made the flavor of dry-cured black carp better by accelerating the oxidation of unsaturated fatty acids. This was because the reaction promotes the formation of volatile flavor compounds such as aldehydes, alcohols and ketones. Recently, Luo, et al. [26] found that CP treatment improved the taste of meat products and analyzed that the improvement of meat product flavor may be due to changes in protein structure and surface, or it may be due to the increased hydrophobicity and hydrophobicity of active oxides produced by CP. The food flavor after CP treatment is closely related to its ingredients. Therefore, the main ingredients (such as proteins and fatty acids) that affect the flavor changes of the food should be clarified before the study to prevent the food from having a peculiar smell [57].

The good news was that studies have reported that using natural extracts (such as chamuang leaf extract, coconut peel extract) to pre-treat the sample before CP treatment can effectively improve the flavor. For example, Olatunde, et al. [58] observed that the effect of seabass slices treated with natural extracts in combination with CP was better than that of CP treatment alone. This phenomenon is attributed to lipid oxidation which gives it an unpleasant odor. Ethanol coconut shell extract is an antioxidant that reduces the lipid oxidation rate and inhibits unpleasant odor in CP-treated Asian sea bass fillets.

## 4. The Effect of CP on Nutrient Content of Foods

Plasma reaction is a complex process which can produce a large number of different kinds of active species. These plasma species are mainly through oxidative degradation and surface etching and other effects on the nutrient content of food. This chapter mainly discusses the impact of CP reactions on certain significant nutrient content.

### 4.1. Proteins

**Figure 3:**
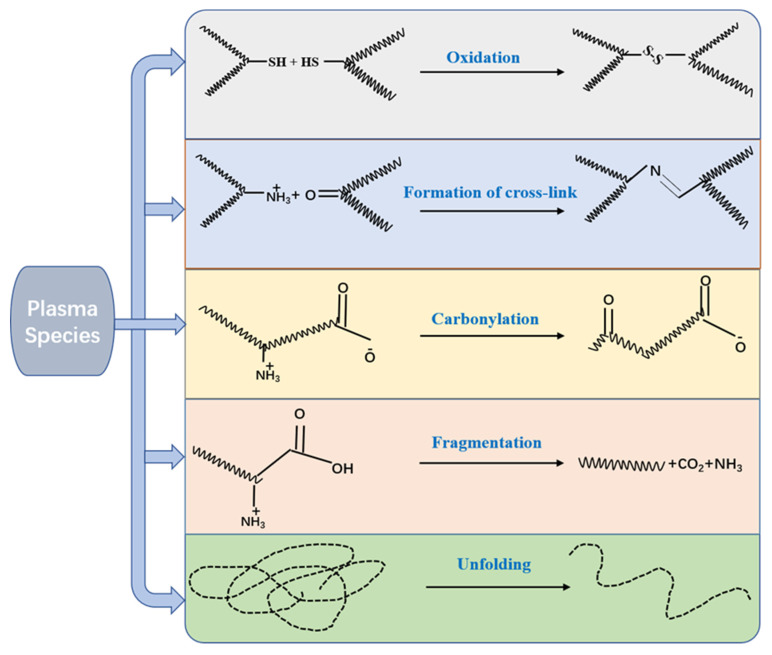
The effect of cold plasma on proteins, adapted from [60].

Proteins are important structural components of food. As indispensable nutrients, they can provide amino acids to the human body. They have a variety of structural and functional properties in the human body, such as emulsifying and gelling abilities, foaming, water and oil holding capacity [59]. Amino acids are the basic units of proteins, and any changes in them will affect the spatial structure of the proteins and thus change the function of the proteins [60]. Figure 3 depicts the effect of CP on amino acids. The chemically active substances produced during the CP reaction, such as active nitrides and active oxides, caused the cleavage of some bonds and chemical modifications on the side chains (such as tryptophan, tyrosine, the aromatic rings of phenylalanine and cysteine), which may cause changes in the protein structure. Even an amino acid change can have a considerable impact on protein function [61]. It has been reported that plasma species can cause a decrease in free sulfide groups and an increase in carbon-based content. According to Segat, et al. [62], when the whey protein isolate was processed at 70 kV within 1–60 min, the carbonyl content in the CP-treated samples was significantly increased, which was due to changes in the amino acid side chain groups, especially with peptide bonds or −NH or −NH_2_. After the DBD system at 60 kV was used to treat crude squid shell protease, the carbonyl group content increased and the free sulfhydryl group decreased for different treatment times [63]. Sharifian, et al. [64] also reported similar results that after DBD plasma treatment, the carbonyl content of beef myofibril protein increased, and the content was higher when the treatment time increased.

After CP treatment, the unfolding and conformational changes of the protein will affect its functional properties. The type and quantity of plasma species generated after CP treatment will affect the interface characteristics of the protein. Furthermore, studies have shown that changes in protein structure and function after CP treatment also depended on the input voltage and current, exposure time, carrier gas type and pressure [65]. Segat, et al. [62] reported that when a whey protein isolate (WPI) was treated with CP, the foaming ability of the protein was improved after 15 min. The better arrangement of proteins at the air–water interface, the unfolding of proteins and alters in structure may account for this phenomenon. As the treatment time increased, the foaming of the whey protein isolate had a negative effect due to the formation of aggregates, and the foaming stability was enhanced by forming a highly rigid film with a strong intermolecular interaction and high bulk density [62]. Recently, Luo, et al. [26] studied the effect of CP treatment on the structure and function of the myofibrillar proteins of meats and the taste of meat products. The results showed that the improvement in the flavor of meat products may be attributed to the changes in protein structure and surface, or it may be due to the active oxides produced by CP that increase the hydrophobicity and the unfolding of myofibrillar proteins. In addition, Zhang, et al. [66] pointed out in the study that a lower CP frequency (80 Hz) has a more positive effect on changing the foaming and emulsification ability of soy protein, and the foaming stability of the sample is the highest at 120 Hz.

The effect of cold plasma on protein in food may be beneficial or harmful, depending on the needs of consumers. A large number of studies on proteins have shown that the extent to which CP changes the structure and function of proteins depends on the characteristics of the protein in the sample (length, branching, compactness of amino acids, etc.) and processing conditions (input voltage and current, exposure time, carrier gas type and its pressure, etc.). Therefore, numerous further studies are needed to obtain the best processing parameters.

### 4.2. Lipids

Lipids oxidation is a worrying problem in foods. It has a negative impact on sample flavor, color, nutritional value and shelf life, and may even cause cardiovascular disease. Peroxide value (PV) and thiobarbituric acid reactive substance (TBARS) are commonly used methods for determining lipid oxidation. The oxidation rate is affected by many factors such as the type of sample matrix, antioxidant or pro-oxidant compounds and the degree of unsaturation [67]. Since the oxide produced by CP treatment has a strong oxidizing effect, it is necessary to analyze the effect of CP on lipids in food. Albertos, et al. [68] reported the effect of CP treatment on lipids in fresh mackerel fillets. In the study, the samples were treated with plasma generated at a voltage of 80 kV for 5 min, and the PV had a significant increase. Recently, Na, et al. [69] studied the degree of oxidation of lipids and the role of antioxidants in plasma treatment. It was found that the conjugated dienoic acid changed significantly after the plasma treatment for 10 min, which corresponds to the treatment at 60 °C for 48 h and the treatment at 100 °C for 2.5 h. The study by Sarangapani, et al. [70] indicated that the treatment of dairy lipids and beef with CP will lead to the oxidation of C=C bonds in unsaturated triglycerides and the generation of new carbonyl compounds (carboxylic acids, saturated aldehydes or other secondary oxidation products). The results also show that the oxidation of lipids caused by cold plasma follows the Criegee mechanism. They found that the typical oxidation products in meat fat and dairy products are ozonide, carboxylic acids (octanoic acid, 9-oxononanoic acid and nonanoic acid) and aldehydes (pentenal, hexanal, nonanal and nonenal) along with hydroperoxides.

Preventing the lipid oxidation of high-fat foods in CP treatment is a challenging task, which may require the optimization of the product and process parameters during CP processing. The pro-oxidant components produced and improper storage conditions will make food lipids more susceptible to oxidation. Using plasma technology in a lower power range, shortening the processing time, adjusting the food ingredients (such as reducing the fat content, adding antioxidants) and choosing the right type of food are all effective ways to overcome lipid oxidation [23].

### 4.3. Phenolic Compounds

Phenolic compounds are important biologically active substances in food that can significantly affect the antioxidant capacity of food and protect food from free radical activities. There are many kinds of phenolic compounds in food, such as anthocyanins, lignans, ellagic acid, phenolic acid, etc. Phenolic compounds have a positive effect on health, and they have a good effect on preventing cancer, coronary heart disease and other diseases. In recent years, many experiments have evaluated the effect of CP on phenolic compounds [71,72,73,74].

Some scholars found that the total phenol content in blueberry juice and apple juice increased significantly after CP treatment [25,33,75]. They analyzed that the increase in phenolic compounds may be due to the decomposition of cell membranes by the active substances produced by CP, which increased the extraction of phenols. Bursać Kovačević, et al. [76] studied the effect of CP on anthocyanins in pomegranate juice. It was found that the anthocyanins were most stable at 3 min of processing time, 0.75 dm^3^/min gas flow and 5 cm^3^ sample volume. The increase in anthocyanin content is related to sample volume and gas flow. This change may also be due to the increased extractability caused by the breakdown of the cell structure, which is usually observed in raw squeezed beverages. The content of the phenolic compounds in the plasma treatment of walnuts and black pepper changed little, which may be attributed to the difference in food structure that limited the penetration of plasma species [71,77]. In general, plasma treatment has a positive effect on the stability of food phenolic compounds. However, once the treatment intensity exceeds a certain value, it may have an adverse effect on phenolic compounds. For example, in studies on fresh-cut pears, chocolate milk and cherry juice, reductions in phenolic compounds were observed [72,73,74]. Therefore, the process conditions should be optimized to achieve the best treatment results.

### 4.4. Carbohydrates

Carbohydrates are essential substances that constitute the structure of cells and can provide energy for life activities. CP treatment can affect the physical and chemical properties of carbohydrates. Studies have found that the use of DBD plasma to treat blueberries increases their sugar content and can slow down the decline in sugar content at an ambient temperature. Furthermore, the time when the sugar content began to decrease was related to the CP treatment time [78]. Research on orange juice found that after CP treatment, fructose decreased, sucrose increased and oligosaccharides degraded [79]. Another study observed similar results after the plasma treatment of cashew apple juice [75]. These authors confirmed that the degradation of fructose and glucose was due to the formation of reactive oxygen species (such as atomic oxygen, hydroxyl radical or singlet oxygen).

**Figure 4:**
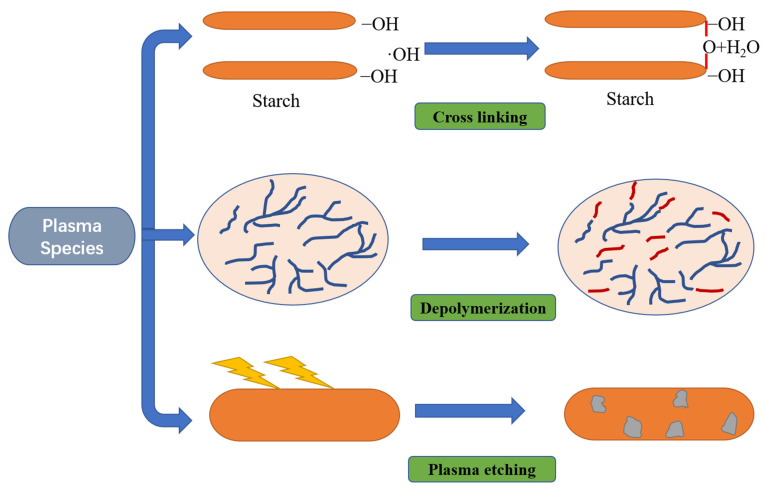
The effect of cold plasma on starch, adapted from [80].

The effects of CP treatment on food polysaccharides are mainly concentrated on starch. CP mainly modifies starch through three mechanisms: depolymerization, cross-linking and plasma etching [80] (Figure 4). Shen, et al. [81] studied the structure, rheology and physicochemical properties of potato starch nanocrystals (SNCs) prepared by CP technology. CP treatment did not alter the crystallization mode and morphology of SNCs but it reduced the crystallinity. The amylose content, gelatinization temperature, swelling power and apparent viscosity of SNCs are reduced after the CP process, which may be attributed to the degradation of SNC molecules and the depolymerization of amylopectin branch chains. Ji, et al. [82] observed that the etching of plasma species changed the microstructure of cassava starch granules, making the surface rough and producing small clumps. With the extension of CP pre-treatment time, the crystallinity of cassava starch decreased. In another study, Thirumdas, et al. [83] studied the effect of CP treatment on rice starch and observed a decrease in the degree of hydrolysis, pasting temperature, gelatinization temperature, degradation tendency and amylose content. In summary, CP treatment can affect the function, structure and rheological properties of starch.

### 4.5. Vitamins

**Figure 5:**
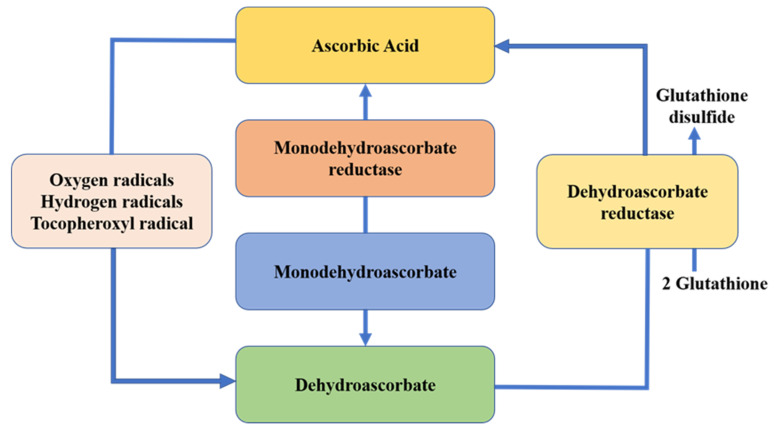
Ascorbic acid decay and regeneration cycle. Adapted from [22].

Vitamins are a trace organic substance that people must obtain from food to maintain normal physiological functions. They play a key role in the growth, development and metabolism of the human body. Some vitamins, such as pyridoxine (B6), riboflavin (B2) and biotin, are relatively stable, while others, such as folic acid (B9) and vitamins E, C and A, are unstable [84]. The decay mechanism of ascorbic acid is through the deprotonation of the molecule to form ascorbate—ascorbate radical—ending in the formation of dehydroascorbate [22] (Figure 5). Some studies have explored the effect of CP technology on the stability of vitamins (especially vitamin C) in food.

Mehta, et al. [85] compared the effects of atmospheric CP with heat treatment, ultraviolet treatment and ultrasonic treatment on vitamin C in tomato beverages and found that the vitamin C retention rate was the highest after 10 min of CP treatment, reaching 95%. The decrease may be due to free radicals or the reactive oxygen generated. In addition, studies have shown that the retention rate of vitamin C was related to the CP treatment time. Xu, et al. [86] processed orange juice for different times under a voltage of 90 kV. The retention rate of vitamin C decreased with the increase in the CP treatment time. When the treatment time was increased to 120 s, the vitamin C in the air was reduced by 22%. Similarly, Hou, et al. [33] noted that when the CP generated by oxygen and argon was applied to process blueberry juice, vitamin C degrades with the increase in oxygen concentration and treatment time. However, other studies have shown the opposite result. Dong, et al. [78] found that CP improved the content of vitamin C in blueberry samples and prolonged its shelf life. The NO generated by plasma can improve the activity of dehydroascorbate reductase, which can reduce oxidized ascorbic acid [87,88]. At low CP flow rates, the regeneration rate of ascorbic acid is greater than the decay rate of its reaction with other plasma-generated reactants, so the vitamin C content increased. Similarly, the CP treatment of cashew apple also observed an increase in vitamin C content [89]. Studies have shown that ascorbic acid can react with the ozone produced in the reaction of CP to reduce its content. Therefore, working conditions that generate large amounts of ozone should be avoided as much as possible when handling vitamin-C-enriched fruit products [22].

Process parameters such as gas type, input voltage and power, exposure time and food characteristics can all affect the degradation of vitamin content. Optimize these process parameters to obtain the most ideal processing conditions to keep the vitamin at a certain level. In short, the effect of plasma treatment on vitamin C in food is more beneficial than harmful. At present, there are few studies on the effects of CP on other types of vitamins, so it is necessary to further study the degradation mechanism of other vitamins by plasma treatment.

## 5. The Effect of CP on Food Safety Performance

In the process of food production, on the one hand, it is necessary to reduce the loss of nutrients, and on the other hand, it is significant to ensure that the output is non-toxic and harmless food. In recent years, many studies have found that the treatment of food with CP technology can degrade allergens and toxins, thereby improving its safety performance [47,90,91].

### 5.1. Microbial Inactivation

CP generation is a very complex physical and chemical reaction process. In this process, active ingredients, charged particles, ultraviolet photons and other bactericidal ingredients are produced. Generally speaking, the good bactericidal effect of CP is the effect of the combined action of these bactericidal substances. The existing research shows that active ingredients play a major role in the sterilization process. Active ingredients mainly include active nitrides and active oxides. These highly oxidizing substances, the phospholipid bilayer of the cell membrane and the peptidoglycan of the cell wall, lead to the rupture of the cell membrane and the cell wall [92]. Existing studies have found that in addition to active species, the ultraviolet photons and charged particles generated during the reaction also have a synergistic sterilization effect. Ultraviolet photons of different wavelengths generated during the CP reaction can damage the amino acid structure of genetic material and proteins in cells [24]. CP also contains a large number of charged particles; a certain amount of charged particles can change the permeability of the cell membrane, make the cytoplasm flow out and lead to cell death [93]. The specific role of each bactericidal component in inhibiting the growth of microorganisms is still controversial, and further research is needed in the future.

Table 2 summarizes the bactericidal effect of CP on solid food and liquid food. Solid and liquid food matrices interact differently with reactive species. CP has a weaker penetration of solid foods. It can only kill some microorganisms on the surface of food. Other conditions being equal, the sterilization effect of solid food can be affected by the shape and surface roughness of the food [94]. Different from solid food, due to the fluidity of liquid, each particle of liquid food can fully contact the active substance generated by CP, and different layers of liquid have the opportunity to be exposed to active substances, so the elimination of CP in liquid is very important. The bacterial depth is greater than the solid surface [95].

### 5.2. Allergens

Food allergies may cause discomfort in the skin, respiratory tract, digestive tract and cardiovascular system. In nature, food allergens are usually glycoproteins or proteins. Heat treatment is a commonly used method to reduce the allergenicity of food, but some allergens retain their antigenicity even after strict heat treatment. In recent years, some studies have shown that CP can reduce food allergens by changing the structure of allergens or destroying the sites where it binds to antibodies.

Ng, et al. [109] explored the effect of CP treatment on the structure and antigenicity of milk proteins (α-lactoprotein, casein and β-lactoglobulin). The results indicated that CP treatment can effectively reduce the antigenicity of α-lactoprotein and casein. However, the antigenicity of β-lactoglobulin has increased. Liu, et al. [110] found that after treatment with plasma gas generated at 40 kV for 5 min, the sensitization and antigenicity of glycine was significantly reduced, and it was shown that these changes were relevant to the cleavage of glycine protein components and protein conformation alteration. Liu, et al. [111] studied the effect of DBD plasma treatment (40 kV, 12 kHz) at different times on the reduction in the immunoglobulin G binding capacity of β-lactoglobulin. The results showed that, compared with untreated samples, the immunoglobulin G binding capacity of β-lactoglobulin gradually decreased after treatment for 1 min and decreased by 58.21% after 4 min of plasma treatment. The decrease in the immunoglobulin G binding capacity of β-lactoglobulin is related to the change in the β-lactoglobulin structure. The initial decline in binding capacity was found to be caused by free sulfhydryl exposure, conformational alteration and the cross-linkage of molecules induced by the oxidation of sensitive amino acid residues and of the NH–/NH2– functional groups of peptide bonds.

### 5.3. Toxins

Toxins pose a huge threat to health. Certain toxins may induce cancer or cause damage to the human nervous system. Studies have shown that CP technology can effectively degrade toxins in food. Hu, et al. [112] evaluated the effect of CP pre-treatment on the formation of polycyclic aromatic hydrocarbons in grilled steak. The results showed that the use of CP technology to pre-treat raw beef before grilling can significantly reduce the production of polycyclic aromatic hydrocarbons during the grilling process. This change is attributed to the improvement of the antiradical activity of raw meat by CP pre-treatment. Shi, et al. [113] reported the effects of gas type, relative humidity and treatment time on aflatoxins in CP-treated corn. The results indicated that under the conditions of a mixed gas (65% O_2_, 5% N_2_, 30% CO_2_) and humid air (80 or 40% RH), the degradation effect of CP treatment on aflatoxin was better than that of air. The mixed gas had a higher content of active species and the humidified air was more likely to produce OH radicals, which is the main reason for the higher yield of aflatoxin degradation. Lee, et al. [114] used corona discharge plasma technology to treat coffee beans and found that the concentration of acrylamide (ACR) and benzopyrene (BaP) decreased after a certain period of time. Another study found that the removal rate of HT-2 and T-2 toxins by the plasma generated by nitrogen as the reaction gas was as high as 38.54% and 43.25%, and the treatment effect was related to time [9].

## 6. Limitations and Future Perspective

The current limitation of CP technology is that its impact on product quality mainly remains at the laboratory stage. The difficulty in the commercialization of CP technology is mainly due to the lack of precise operating conditions and extensive research on the quality characteristics of different foods [21]. There are many variables in the CP processing process, and any change in any variable will have different effects on food quality. Therefore, a large amount of experimental data collection and analysis are needed to optimize the CP process parameters and expand its application range in the food industry. In addition, due to relatively high equipment costs and complicated operation and maintenance procedures, the actual application of CP is also limited.

Numerous different types of active substances are produced during the CP reaction process. The changes in food quality characteristics may be the result of the joint action of multiple active substances, and different plasma species may have different effects on food. We need to further study its reaction mechanism to optimize its reaction conditions. The safety of CP technology is also a crucial issue. In terms of safety, comprehensive research is needed to ensure that substances which cause serious damage to human health will not be produced during its reaction. In addition, CP technology can also be combined with other emerging non-thermal processing technologies to make up for the shortcomings of CP technology to achieve the best results.

## 7. Conclusions

As a new and green non-thermal treatment technology, CP can effectively improve food quality and extend shelf life. In recent years, there have been many studies on the application of CP in the purification of food microorganisms, the degradation of pesticides and toxins in food and the surface treatment of food packaging materials. However, there is little literature on the effect of CP treatment on the functional composition and quality characteristics of food. Studies have found that the influence of plasma on the quality of food is mainly attributed to the active species produced during CP reaction. Through surface etching, oxidative degradation, starch depolymerization and other mechanisms of action, these plasma species can change organoleptic properties (color, taste and texture) and nutrient content (such as proteins, lipids, carbohydrates, phenolic compounds and vitamins). In terms of food safety performance, CP can not only kill spoilage microorganisms, but also degrade allergens and toxins in food. In general, CP treatment has both favorable and unfavorable effects on product attributes. The degree of influence on food characteristics is closely related to factors such as exposure time, carrier gas type, input voltage and the food matrix. Therefore, we must optimize the process parameters as much as possible to avoid the adverse effects of CP treatment on food, such as accelerated lipid oxidation, the deterioration of organoleptic properties, loss of vitamins, carbohydrates and certain beneficial proteins. In the future, we must have more extensive research and a more precise understanding of the mechanism of action between CP species and food ingredients, so that CP technology has a wide range of applications in the food industry.

## Figures and Tables

**Table 1:** Effect of cold plasma treatment on organoleptic properties of various food products.

Food Status	Food Products	Plasma	Treatment Conditions	Quality Observation	Reason for Change	References
Liquid	Tomato juice	DBD	10 kHz; 0–5 min; Air	with the increase in treatment time, the difference in total color value of tomato juice increased significantly; Browning index increased from 2.35 to 4.54	The decomposition of carotenoid pigments by plasma species	[31]
	Apple juice	Electric discharge plasma	15, 18, 21 kV; 50 Hz; 0–30 min; Air	The hue value and brightness of apple juice are increased, the juice became brighter and more yellow	Oxidation reaction and pigment isomerization	[32]
	Blueberry juice	Jet plasma	11 kV; 1000 Hz; 2–6 min; Ar and O_2_	CP treatment better preserves original color of blueberry juice	_	[33]
	Siriguela juice	GDP	80 W; 50 Hz 5–15 min; N_2_	Color and flavor of Siriguela juice did not change significantly with increased processing time	_	[34]
Solid	Strawberries	DBD	100 kV; 2.5 min	The color and firmness of strawberries vary slightly	CP treatment inhibits microbial growth on strawberries	[35]
	Metmyoglobin and Oxymyoglobin	PAS	60 V; 10 kHz; 0.5–10 min	Metmyoglobin faded first and then turned green; oxymyoglobin changed from bright red to red and finally to green	The accumulation of active species of H_2_O_2_ in PAS	[36]
	Pork jerky	DBD	3.8 kV; 4 kHz; 0–60 min; Air	Jerky becomes brighter in color, more red and less yellow	CP treatment promotes nitrite production	[37]
	Hairtail	ACP	50 kV; 30–300 s; Air	Brightness values, elasticity, co-hesion and stiffness increased	The increase in water holding capacity; oxidizing hairtail muscle proteins	[38]
	Shiitake mushrooms	PAW and DBD	1200 W; 20 min; N_2_ and O_2_	The brightness value was higher than that of the control sample After 1 week of storage, increased in hardness value and the PAW mushroom had the highest hardness value	Inactivation of deteriorative enzymes; reduction in bacterial load	[39]
	Corn Kernels	DBD	40 kHz; 300, 400, 500 W; 30–50 s	The topography of the treated surface has changed	Etching effect of CP species	[40]
	Chili peppers	GAD	20 kHz, 750 W; 15–60 s;	No significant change in color at different treatment times; the surface dried faster	Etching effect of CP species	[41]
	Almond slices	APPJ	17 V; 5–20 min; Ar	Increased firmness of almond slices	CP species react with moisture in almond slices	[42]
	Fresh wet noodles	DBD	100 kHz; Air	The color turns white and the texture becomes hard	CP species react with moisture in noodles	[43]
	Bayberries	PAW	20 kHz; 0.5–5 min	The color changes from red or purple to dark purple. Texture maintains firmness	Oxidation of plasma species; fungus suppressed on Bayberries	[44]
	Dried filefish fillets	COP	0–20 min; Air	After 20 min, the flavor value was significantly reduced	CP species induced lipid oxidation	[45]
	Asian sea bass slices	DBD	230 V; 50 Hz; 5 min; Ar and O_2_	The Asian sea bass fillets produce peculiar odor after CP treatment and reduce overall acceptance	The increased lipid oxidation rate of plasma species	[46]

Note: DBD = dielectric barrier discharge; CD = corona discharge; PAW = plasma activated water; GAD = gliding arc discharge; APPJ = atmospheric pressure plasma jet; COP = cold oxygen plasma; ACP = atmospheric cold plasma; Ar: argon; N_2_: nitrogen; O_2_: oxygen.

**Table 2:** Recent findings on microbial inactivation of various food products using CP technology.

Food Status	Food	Plasma	Treatment Conditions	Microbial Reduction	References
Solid	Raw chicken breast	DBD	55 kV; 2.5 min; Air	1.1 lg CFU/mL *Campylobacter jejuni* 0.3 lg CFU/mL *Salmonella typhimurium*	[96]
	Cherry tomatoes	DBD	35 kV; 1.1 A; 3 min	0.9 lg CFU/sample *Salmonella*	[97]
	Cured beef	APPJ	25 kV; 42 kHz; 5 min; Ar and O_2_	0.85 lg CFU/cm^2^ *Staphylococcus aureus* 0.85 lg CFU/cm^2^ *Listeria monocytogenes*	[98]
	Blueberry	DBD	12 kV; 5 kHz; 1 min	0.57~0.87 lg CFU/g mold 0.34~1.24 lg CFU/g(P < 0.05) total aerobic bacteria	[99]
	Oyster mushroom	DBD	6 kV; 25 min; Air	0.8 lg CFU/mL colony	[100]
	Korean rice cake	PAW	51.7 W; 14.4 kHz; 20 min; Air	2.01–2.03 lg CFU/g *Escherichia coli* 2.08–2.12 lg CFU/g *Salmonella typhimurium* 1.98–2.17 lg CFU/g *Listeria monocytogenes*	[101]
	Fresh cut carrot	Cold atmospheric plasma	100 kV; 60 Hz; 5 min; Air	2.1 lg CFU/g mesophiles and yeast	[102]
	Mandarins	DBD	27 kV; 2 min	Reduction in *Penicillium digitatum* disease	[103]
	Ricotta cheese	DBD	13.8 kV; 6 kHz; 5 min; N_2_ and O_2_	Reduction in *Pseudomonas* and *Enterobacteriaceae*	[104]
Liquid	Apple juice	APPJ	25 kHz; 650 W; 2 min; Air	4.02 lg CFU/mL *Escherichia coli*	[105]
	Milk	DBD	250 W; 15 kHz; 10 min; Air	2.46 lg CFU/mL *Salmonella typhimurium* 2.43 lg CFU/mL *Escherichia coli* 2.40 lg CFU/mL *Listeria monocytogenes*	[106]
	Cherry juice	APPJ	25 kHz; 650 W; 2 min; Air	3.34 lg CFU/mL *Escherichia coli*	[105]
	Coconut milk	DBD	90 kV; 2 min; Air	1.30 lg CFU/mL *Salmonella*	[107]
	Rose water	CD	100 W; 45 S; Ar	Inactivation of mold and yeast cells is reduced by around 70%	[108]

Note: DBD = dielectric barrier discharge; CD = corona discharge; PAW = plasma activated water; GAD = gliding arc discharge; APPJ = atmospheric pressure plasma jet; COP = cold oxygen plasma; ACP = atmospheric cold plasma; Ar: argon; N_2_: nitrogen; O_2_: oxygen.

## Data Availability

All data is publicly available.
